# A Retrospective Analysis: CCTA *vs.* TTE in Diagnosing Coronary Artery Fistula

**DOI:** 10.2174/0115734056333289250311184106

**Published:** 2025-04-17

**Authors:** XiaoLi Hu, Jian Shen, ChunLi He, YueMei Li, Shen Gui, YuKun Cao, Ping Han, Jun Xu

**Affiliations:** 1 Department of Radiology, Wuhan Asia Heart Hospital, Wuhan 430022, China; 2 Department of Hepatic, Biliary and Pancreatic Surgery, Hubei Cancer Hospital, Tongji Medical College, Huazhong University of Science and Technology, Wuhan 430079, China; 3 Department of Ultrasound, Wuhan Asia Heart Hospital, Wuhan 430022, China; 4 Clinical Science, Philips Healthcare, Wuhan, China; 5 Department of Radiology, Union Hospital, Tongji Medical College, Huazhong University of Science and Technology, Wuhan 430022, China; 6 Hubei Provincial Clinical Research Center for Precision Radiology & Interventional Medicine, Wuhan 430022, China; 7 Hubei Key Laboratory of Molecular Imaging, Wuhan 430022, China

**Keywords:** Cardiac computed tomographic angiography (CCTA), Coronary artery fistula (CAF), Imaging diagnosis, Transthoracic echocardiography (TTE), Vascular anomaly, Digital subtraction

## Abstract

**Objective:**

This study aimed to compare and analyze the diagnostic performance of cardiac computed tomographic angiography (CCTA) and transthoracic echocardiography (TTE) for coronary artery fistula (CAF) and evaluate the effectiveness of these two imaging modalities.

**Methods:**

We retrospectively collected and analyzed imaging data from 200 patients diagnosed with CAF through surgery or digital subtraction angiography (DSA). These patients underwent CCTA and TTE examinations in our hospital. Finally, the course, origin, number, size, and location of the CAF in all patients were assessed. The diagnostic results of CCTA were compared with those of TTE, using DSA and/or surgical diagnosis as the reference standard.

**Results:**

Among the 200 patients with CAF, CCTA correctly diagnosed 156 cases, but missed 44 cases, resulting in a diagnostic accuracy of 78.0% (156/200). In contrast, TTE accurately diagnosed 55 cases, but missed 145 cases, yielding a diagnostic accuracy of 27.5% (55/200). The diagnostic accuracy of CCTA was significantly higher than that of TTE in detecting CAF (*P* < 0.001).

**Conclusion:**

CCTA demonstrated significantly greater diagnostic value than TTE, demonstrating to be the preferred imaging modality for diagnosing CAF.

## 1 INTRODUCTION

Coronary artery fistula (CAF) is a rare vascular anomaly in clinical practice (with an incidence rate of 0.5% or even lower) [1, 2]. It is characterized by an abnormal connection between the coronary artery trunk or its branches and the cardiac chambers or major blood vessels [3-6]. The clinical presentation of CAF lacks specificity, and some patients may present with symptoms, such as dyspnea, chest pain, and palpitations. In more severe cases, CAF can significantly impair myocardial perfusion, potentially resulting in myocardial ischemia or even sudden death [7, 8]. Therefore, early and accurate diagnosis of CAF is crucial.

There are multiple imaging methods available for the clinical diagnosis of CAF, including digital subtraction angiography (DSA), cardiac computed tomographic angiography (CCTA), transthoracic echocardiography (TTE), and magnetic resonance coronary angiography (MRCA). DSA, regarded as the “gold standard”, enables direct visualization of coronary artery anatomy, the morphology of the coronary artery and its branches, as well as the connections with cardiac chambers or blood vessels. However, this method is invasive and entails the risk of complications and high radiation exposure [9, 10]. MRCA offers advantages, such as multiparametric, multisequence, and multiplanar imaging, along with being radiation-free. However, this approach is constrained by relatively slow image acquisition, susceptibility to motion artifacts induced by breathing and cardiac motion, and inadequate visualization of small and tortuous fistulae, thereby restricting its clinical application [11-14]. In recent years, CCTA has gained widespread clinical use due to its high temporal and spatial resolution, low radiation dose, and good image quality. It offers a noninvasive option for assessing coronary artery lesions and providing preoperative planning for cardiac interventions. Additionally, with advancements in ultrasound technology, TTE has become another non-invasive diagnostic method for identifying CAF. TTE not only provides detailed real-time dynamic visualization of CAF blood flow, but also offers strong repeatability, no radiation exposure, and lower cost.

Therefore, the non-invasive examination methods for clinical diagnosis of CAF are mainly CCTA and TTE. Although two studies have explored the diagnostic performance of CCTA and TTE for CAF, there have been differences in the research results, and the sample sizes have not been more than 60 cases [15, 16]. Given that CAF is a rare vascular abnormality and large-sample imaging studies are currently scarce, this study collected data from 200 CAF patients diagnosed by surgery or DSA. The aim was to compare and analyze the imaging diagnostic results of CCTA and TTE, and explore the diagnostic efficacy of these two methods in identifying CAF.

## 2 MATERIALS AND METHODS

### 2.1 Patient Population

Patients who were diagnosed with CAF and admitted to the Asia Heart Hospital from January 2016 to November 2021 were retrospectively analyzed in this study. The study flowchart is shown in Fig. (**1**). The inclusion criteria for patients were as follows: 1) the diagnosis confirmed by surgery and/or DSA; 2) undertaken both CCTA and TTE examinations at our hospital within three months; 3) availability of complete and comprehensive clinical case data and imaging results. The exclusion criteria were as follows: 1) the diagnosis not confirmed by surgery and/or DSA; 2) only one type of examination (either CCTA or TTE) undertaken within three months; 3) incomplete or poor-quality imaging information obtained for both CCTA and TTE examinations. This study was approved by the ethics committee of the Asia Heart Hospital (ethics no. 2022-13031) and all patients signed an informed consent form.

### 2.2 CCTA Examination Method

Before scanning, patients were provided with respiratory training and instructed to place nitroglycerin tablets under their tongues. A Brilliance iCT scanner (Netherlands, Philips) or a Somatom Definition FLASH CT scanner (Siemens, Germany) was used, with electrocardiogram gating and intelligent tracking triggering scan technology. The region of interest (ROI) was set at the root of the aorta, and the scanning commenced when the aortic CT value reached 120-140 Hounsfield units (HU). The scan covered the area from the tracheal bifurcation to 2 cm below the cardiac diaphragm. The scanning parameters of the two devices were identical: a tube voltage of 100-140 kV, intelligent milliampere-seconds (mAs) for dose modulation, a pitch automatically adjusted based on heart rate, a slice thickness of 0.60-0.75 mm, an interlayer spacing of 0.3-0.4 mm, and a matrix of 512×512. After the examination, the best diastolic and systolic coronary artery images were reconstructed and uploaded to the Picture Archiving and Communication System (PACS) and postprocessing workstation.

### 2.3 TTE Examination Method

The patients were positioned either in the supine or in the left lateral position during the examination. A color Doppler ultrasound diagnostic instrument (Aloka Prosound F75, Hitachi, Japan) was used to continuously observe and assess the presence of coronary artery dilation, tortuosity, and abnormal blood flow signals within the cardiac chambers and major vessels; the origin, morphology, and course of the fistula; as well as the location, size, and number of feeding vessels in the fistula.

### 2.4 Imaging Evaluation

Two experienced cardiovascular imaging specialists from the radiology department and two from the ultrasound department independently diagnosed and evaluated the course, origin, number, size, and location of the CAF in all patients. If there was a disagreement between the two doctors within their respective departments, the assessment was reviewed by senior professionals in their field to reach a consensus. The diagnostic results of each department were then obtained. Finally, taking DSA and/or surgical diagnosis as the reference standard, the diagnostic results of CCTA were compared with those of TTE.

### 2.5 Statistical Analysis

The obtained data were analysed using the statistical software SPSS (Version 26.0, IBM America GmbH, USA), and a chi-square test was conducted to evaluate the diagnostic accuracy of the two imaging methods. The diagnostic accuracy was estimated as follows: (the number of detected cases/the total number of cases)×100%. The continuous data have been presented as mean ± SD. The count data were expressed as the number or number (percentage), namely n or n (%). *P*<0.001 indicated statistical significance.

## 3 RESULTS

### 3.1 General Information

A total of 798 patients with clinically suspected CAF were screened in the hospital's case management system. Based on the inclusion and exclusion criteria, 200 patients (100 males and 100 females; age range: 0.08-81 years; mean age: 49.68±23.14 years) with CAF were ultimately included. The clinical characteristics of all patients with CAF are shown in Table **1**.

### 3.2 DSA and/or Surgery Results

Among the 200 patients with CAF, 1) 65 patients were diagnosed via surgical procedures. Among them, 64 patients underwent surgical closure, and only 1 patient incidentally discovered the CAF during surgery for another cardiac condition, but did not receive treatment due to the small size of the fistula. 2) 135 patients were diagnosed with CAF through DSA. Among these patients, 50 patients underwent transcatheter closure, 13 patients underwent surgical closure for concomitant other cardiac conditions after the CAF was identified through DSA, and 72 patients did not undergo surgical treatment. Therefore, 50 (25.0%) patients underwent transcatheter closure, 77 (38.5%) patients underwent surgical closure, and 73 (36.5%) patients did not receive treatment.

### 3.3 Classification of CAFs

As shown in Table **2**, 1) classification based on origin included 73 patients (36.5%) having left CAF, 59 patients (29.5%) having right CAF, and 68 patients (34%) having both left and right CAF. 2) Classification based on drainage site included 126 patients (63%) having coronary artery-pulmonary artery fistulas, 44 patients (22.0%) having coronary artery-ventricle fistulas, 24 patients (12%) having coronary artery-atrium fistulas, 3 patients (1.5%) having coronary artery-coronary sinus fistulas, 2 patients (1%) having coronary artery-bronchial artery fistulas, and 1 patient (0.5%) having both coronary artery and bronchial artery-pulmonary artery fistulas. 3) Classification based on fistula size included 50 patients (25.0%) having small fistulas, 87 patients (43.5%) having medium fistulas, and 63 patients (31.5%) having large fistulas. 4) Classification based on blood shunt direction included 174 patients (87.0%) having left-to-right shunting and 26 patients (13.0%) having left-to-left shunting.

### 3.4 Comparison of the Diagnostic Results

**Fig. (2):**
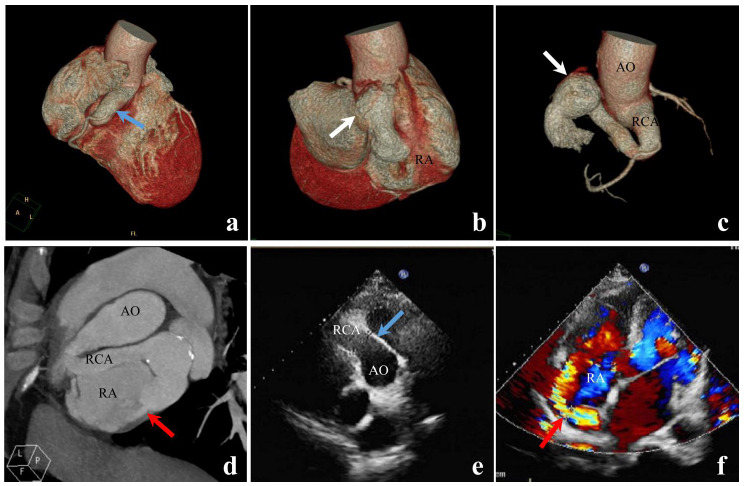
A case of right coronary artery-to-right atrium fistula. Both CCTA and TTE accurately diagnosed the patient’s condition and identified the origin and drainage site of the fistula. The CCTA images visually and accurately illustrate the dilated right coronary artery (**a**, **blue arrow**), the arterial aneurysm (**b** and **c**, **white arrows**), and the fistula draining into the right atrium with the “ejection sign” (**d**, **red arrow**). TTE demonstrates the widening of the right coronary artery at its origin (**e**, **blue arrow**) and abnormal blood flow into the right atrium (**f**, **red arrow**). **a-c**, CCTA volume-rendering (VR) images. **d**, CCTA maximum intensity projection (MIP) image. **e-f**, TTE two-dimensional images. **Note:** AO, aorta; RCA, right coronary artery; RA, right atrium.

**Fig. (3):**
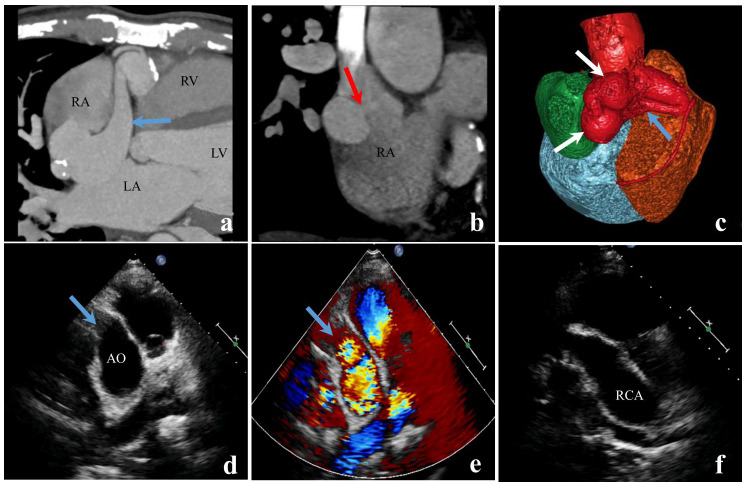
A case of right coronary artery-to-right atrium fistula. CCTA accurately confirmed the existence of this fistula, while TTE identified the fusiform dilation in the proximal part of the right coronary artery, but it was unable to clearly define the course or the final drainage site of the dilated artery. The CCTA image visually and accurately depicts the dilated and tortuous course of the right coronary artery **(a** and **c**, **blue arrows**), the arterial aneurysm (**c**, **white arrow**), and the fistula draining into the right atrium (**b**, **red arrow**). TTE shows the right coronary artery with widening at the origin (**d** and **e**, **blue arrows**), a downward course, and overall dilatation within the explored range. **a,** The CCTA MIP image. **b,** CCTA multiplanar reformation (MPR) image. **c,** CCTA VR image. **d-f,** TTE two-dimensional images. **Note:** AO: aorta; RCA: right coronary artery; RA: right atrium; RV: right ventricle; LA: left atrium; LV: left ventricle.

Among the 200 patients with CAF, CCTA accurately diagnosed 156 cases, but missed 44 cases, exhibiting a diagnostic accuracy of 78.0% (156/200). Of these accurately diagnosed cases, 144 patients had accurate diagnoses of both the origin and drainage site, while in 12 patients, the origin or drainage site of the fistula could not be determined. In contrast, TTE correctly diagnosed 55 cases, but missed 145 cases, resulting in an accuracy of 27.5% (55/200). Within this group, 42 patients had accurate diagnoses of both the origin and drainage site, while in 13 patients, the origin or drainage site of the fistula could not be determined. There was a significant difference in the accuracy of CAF diagnosis between the two imaging methods (*P*<0.001) (Table **3** and Figs. **2** and **3**). Furthermore, both CCTA and TTE had higher diagnostic accuracy for large fistulas than for small-to-medium-sized fistulas, with small-to-medium-sized fistulas being more prone to missed diagnoses (Table **2**).

## 4 DISCUSSION

In this study, the accuracy rate of CCTA was 78.0%, while that of TTE was relatively lower, being 27.5%. The results indicated the diagnostic efficacy of CCTA in identifying CAF to be significantly higher than that of TTE.

TTE presents several advantages, including noninvasiveness, convenience, affordability, real-time dynamic observation, and strong repeatability. Color Doppler ultrasound can reveal the origin and drainage site of the CAF, as well as the diameter of the fistula, the size of the shunt, and the blood flow velocity. This renders it a valuable tool for the preoperative detection, intraoperative exploration, and postoperative follow-up of CAF [17]. TTE is also capable of revealing various related congenital and acquired cardiac defects [18]. However, this technique is significantly affected by patient body size, possesses limited acoustic windows, is highly reliant on operator skills, and cannot fully display dilated fistulas. It lacks the capacity to provide a three-dimensional visualization of CAF and lacks intuitiveness. This limitation becomes especially conspicuous when evaluating small, tortuous fistulas, distal fistulae, or multiple fistulas. Specifically, CAFs that drain into the pulmonary artery are prone to being overlooked due to their small fistula size, absence of significant dilation, and minimal shunt flow [19]. In this study, TTE exhibited the highest number of missed diagnoses for coronary artery-pulmonary artery fistulas, with 113 cases missed, constituting 77.9% (113/145) of all missed cases.

CCTA provides high spatial and temporal resolution, as well as powerful post-processing techniques, such as maximum intensity projection (MIP), multiplanar reformation (MPR), curved planar reformation (CPR), and volume rendering (VR). These capabilities allow for a comprehensive evaluation of CAF from different perspectives, including the origin, drainage site, complexity, and characteristics of the fistula, such as its course, morphology, number, and size [20]. However, suboptimal image acquisition resulting from factors, like patient discomfort (for instance, anxiety or arrhythmia), the existence of multiple calcifications, myocardial bridging, or small fistulas obscured by other structures, can lead to misdiagnosis [21]. In this study, the accuracy of CCTA for diagnosing CAF was 78.0% (156/200), with 44 cases missing. The principal reasons for missed diagnoses were the small size, tortuous course, and complexity of the fistulas or the presence of extremely small drainage sites that were difficult to visualize, constituting 63.6% (28/44) of all missed cases. Additionally, poor image quality, such as motion artifacts caused by cardiac pulsation, resulted in blurry visualization of the fistula course and constituted 15.9% (7/44) of all missed cases.

Previous research studies have explored similar topics. Ouchi *et al*. [15] conducted TTE examinations on 52 patients with CAF. Color Doppler ultrasound detected abnormal blood flow in these patients, with 12 patients suggesting the presence of CAF, resulting in an accuracy of 23.1%. These results align with our findings, indicating that TTE has limited diagnostic value for identifying CAF. Conversely, Xie Mingxing and colleagues [16] conducted a retrospective study on 63 patients who underwent TTE before DSA and/or surgery. They reported a diagnostic concordance with surgery and/or DSA results in 60 patients, achieving an accuracy of 95.2%. This contrasts significantly with our study's conclusions, and the discrepancy may be attributed to differences in sample size, technical proficiency, and operator experience. While studies specifically evaluating the diagnostic value of CCTA for CAF are currently scarce, some studies indicate that CCTA can provide a comprehensive evaluation of complex blood vessels and associated cardiac abnormalities. It is considered a preferred non-invasive imaging method for detecting detailed anatomical information regarding concomitant congenital heart malformations, proving effective, accurate, and diagnostically reliable [22-24].

In clinical practice, accurate diagnosis of CAF is of crucial importance for formulating treatment strategies. The abundant information provided by CCTA helps doctors comprehensively understand the lesion conditions, enabling them to select treatment options more precisely, such as determining whether transcatheter closure or surgical treatment is appropriate and planning the surgical approach. In addition, CCTA also has significant value in postoperative follow-up. It can clearly display the position of the occluder and the situation of residual shunt, and promptly detect and handle possible complications [20, 25].

This study has involved several limitations. First, it was a single-center study. Second, due to the lack of negative cases, the evaluation of the two diagnostic methods was limited. In future research, the experimental design will be refined to conduct a more comprehensive assessment of their diagnostic performance. Third, due to the late development of MRCA, there exists a lack of research on the diagnostic value of MRCA for identifying CAF. Fourth, transesophageal echocardiography can obtain clearer images and help improve the detection rate of CAF. However, it has the risk of invasiveness and thus was not considered in this study.

## CONCLUSION

In conclusion, CCTA offers several advantages, such as rapid and clear imaging, non-invasiveness, and convenience of follow-up. It can provide the spatial structure and anatomical details of CAF, along with information on other concurrent heart diseases. Its diagnostic value is significantly higher than that of TTE, making it the preferred examination method for diagnosing CAF. This may potentially lead to corresponding adjustments in the clinical guidelines for the diagnosis and treatment process of CAF, including preoperative diagnosis and evaluation, formulation of surgical plans, and postoperative follow-up assessment.

## AUTHORS’ CONTRIBUTIONS

The authors confirm their contribution to the paper as follows: study conception and design: CLH, PH, JX; data collection: YKC, YML; analysis and interpretation of results: SG; drafting of the manuscript: XLH, JS. All authors have reviewed the results and approved the final version of the manuscript.

## Figures and Tables

**Fig. (1):**
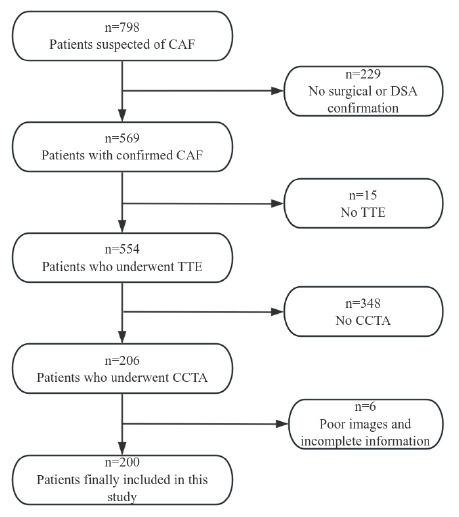
Flowchart of the inclusion and exclusion criteria. **Note:** CAF, coronary artery fistula; CCTA, cardiac computed tomographic angiography; DSA, digital subtraction angiography; TTE, transthoracic echocardiography.

**Table 1:** Clinical characteristics of 200 patients with CAF.

Clinical Characteristics	n (%)
**Sex**	
Male	100 (50.0)
Female	100 (50.0)
Mean age (years)	49.68±23.14
Age range (years)	0.08-81
**Age Groups**	
0-9 years	28 (14.0)
10-24 years	6 (3.0)
25-49 years	25 (12.5)
50-74 years	127 (63.5)
≥75 years	14 (7.0)
BMI (kg/m2)	23.28±4.90
**Complications**	
Hypertension	74 (37.0)
Diabetes mellitus	21 (10.5)
Dyslipidemia	31 (15.5)
Previous MI	10 (0.5)
Known CHD	127 (63.5)
Arrhythmia	55 (27.5)
Other congenital cardiac malformations	19 (9.5)
**Clinical Symptoms**	
Asymptomatic	57 (28.5)
Symptomatic*	143 (71.5)
**Treatments**	
Transcatheter interventional	50 (25.0)
Surgery	77 (38.5)
Conservative treatment	73 (36.5)

BMI, body mass index; MI, myocardial infarction; CHD, coronary heart disease. *Symptoms included dyspnea, thoracalgia, oppression in the chest, chest tightness, and palpitation.

**Table 2:** The diagnostic results of CCTA and TTE in different CAFs (200 cases).

**CAF Categories**	**Total (cases)**	**CCTA**			**TTE**		
		**Detected (cases)**	**Undetected (cases)**	**Inaccurate localization in Origin/drainage Site (cases)**	**Detected (cases)**	**Undetected (cases)**	**Inaccurate Localization in Origin/drainage Site (cases)**
**Origin**							
LCA	73	50	23	1	15	58	5
RCA	59	48	11	1	31	28	1
LCA and RCA	68	58	10	10	9	59	7
**Drainage site**							
PA	127	96	31	11	13	114	11
RA	20	16	4	0	14	6	1
RV	24	22	2	0	18	6	0
LA	4	2	2	0	1	3	0
LV	20	17	3	1	7	13	1
CS	3	3	0	0	2	1	0
BA	2	0	2	0	0	2	0
**Fistula diameter(D:mm)**							
Small(D≤2)	50	32	18	1	7	43	2
Medium(2<D≤4)	87	68	19	8	18	69	6
Large(D>4)	63	56	7	3	30	33	5
**Shunt direction**							
Left to right	174	137	37	11	47	127	12
Left to left	26	19	7	1	8	18	1

LCA: left coronary artery, RCA: right coronary artery, PA: pulmonary artery, RA: right atrium, RV: right ventricle, LA: left atrium, LV: left ventricle, CS: coronary sinus, BA: bronchial artery.

**Table 3:** The diagnostic accuracy of CCTA and TTE.

Imaging Methods	Detected (cases)	Undetected (cases)	Diagnostic accuracy	*χ* ^2^-value	*P*-value
CCTA	156	44	78.0%	101.00	<0.001
TTE	55	145	27.5%		

The diagnostic accuracy was estimated as follows: (the number of detected cases/the total number of cases)×100%.

## Data Availability

The data and supportive information are available within the article.

## LIST OF ABBREVIATIONS

CAF: Coronary artery fistula
CT: Computed tomography
CCTA: Cardiac computed tomographic angiography
CPR: Curved planar reformation
DSA: Digital subtraction angiography
HU: Hounsfield unit
MIP: Maximum intensity projection
MPR: Multiplanar reformation
ROI: Region of interest
TTE: Transthoracic echocardiography
VR: Volume rendering

## STANDARDS OF REPORTING

STROBE guidelines were followed.
